# Risk and protective factors associated with mental health status in an Italian sample of students during the fourth wave of COVID-19 pandemic

**DOI:** 10.1186/s13034-023-00615-w

**Published:** 2023-06-26

**Authors:** Mariangela Lanfredi, Jessica Dagani, Andrea Geviti, Federica Di Cosimo, Maria Bussolati, Luciana Rillosi, Donatella Albini, Marina Pizzi, Roberta Ghidoni, Elisa Fazzi, Antonio Vita, Roberta Rossi

**Affiliations:** 1 Unit of Psychiatry, IRCCS Istituto Centro San Giovanni di Dio Fatebenefratelli, Via Pilastroni 4, 25125 Brescia, Italy; 2 SIPEC Foundation, Brescia, Italy; 3 Department of Clinical and Experimental Sciences, Section of Clinical and Dynamic Psychology, University of Brescia, Brescia, Italy; 4 Service of Statistics, IRCCS Istituto Centro San Giovanni di Dio Fatebenefratelli, Brescia, Italy; 5 USR Lombardia, Ufficio IV Ambito Territoriale di Brescia, Brescia, Italy; 6 Associazione ITACA Brescia, Brescia, Italy; 7 Comune di Brescia, Brescia, Italy; 8 Department of Molecular and Translational Medicine, University of Brescia, Brescia, Italy; 9 Molecular Markers Laboratory, IRCCS Istituto Centro San Giovanni di Dio Fatebenefratelli, Brescia, Italy; 10 Child Neurology and Psychiatry Unit, Spedali Civili Hospital, Brescia, Italy; 11 Department of Clinical and Experimental Sciences, University of Brescia, Brescia, Italy; 12 Department of Mental Health and Addiction Services, Spedali Civili Hospital, Brescia, Italy

**Keywords:** COVID-19, Mental health, Adolescents, Young adults, Psychological distress

## Abstract

**Background:**

It is well known that the COVID-19 pandemic has caused a global health crisis, especially for young people. However, most studies were conducted during the first waves of the pandemic. Few Italian studies specifically attempted to broadly assess young people’s mental health status during the fourth wave of the pandemic.

**Methods:**

This study aimed at evaluating the mental health status among a group of Italian adolescents and young adults during the fourth wave of the COVID-19 pandemic. 11,839 high school students and 15,000 university students (age range 14–25) were asked to complete a multidimensional online survey, of which 7,146 (26,6%) agreed to participate. The survey also included standardized measures for depression, anxiety, anger, somatic symptoms, resilience, loneliness and post-traumatic growth. Two separate clusters were identified through cluster analysis. Random forest, classification tree and logistic regressions analyses were applied to identify factors associated to a good or a poor level of mental health and, thus, to define students’ mental health profiles.

**Results:**

Overall, the students in our sample showed high levels of psychopathology. The clustering methods performed identified two separate clusters reflecting groups of students with different psychological features, that we further defined as "poor mental health" and "good mental health". The random forest and the logistic regressions found that the most discriminating variables among those two groups were: UCLA Loneliness Scale score, self-harm behaviors, Connor-Davidson Resilience Scale-10 score, satisfaction with family relationships, Fear of COVID-19 Scale score, gender and binge eating behaviors. The classification tree analysis identified students’ profiles, showing that, globally, poor mental health was defined by higher scores of loneliness and self-harm, followed by being of female gender, presenting binge eating behaviors and, finally, having unsatisfying family relationships.

**Conclusions:**

The results of this study confirmed the major psychological distress caused by the COVID-19 pandemic in a large sample of Italian students, and provided further insights regarding those factors associated with a good or poor mental health status. Our findings suggest the importance of implementing programs targeting aspects that have been found to be associated to a good mental health.

**Supplementary Information:**

The online version contains supplementary material available at 10.1186/s13034-023-00615-w.

## Background

The coronavirus disease (COVID-19) was officially declared as a pandemic in March 2020 and caused a global health crisis. Italy was the first European country severely hit by the pandemic. Since then, there have been four major epidemic waves. In Italy, the last major wave started in August 2021 and continued throughout the same year. The containment measures put in place by the government to limit the spread of the virus were mainly based on quarantine and social distancing, including lockdowns and school closures, which strongly limited social interactions and caused great disruption in the daily lives of millions of people (especially young people), as well as having a strong impact on family dynamics. Although these measures have been effective in preventing the spread of the virus, some concerns regarding the psychological impact of isolation have been arisen [81].

So far, studies and surveys conducted during the pandemic consistently show the devastating impact on the general population’s mental health [27, 61, 70] and the greater vulnerability of young people to increased psychological distress, perhaps because their need for social interactions is stronger [1]. Indeed, data confirmed high levels of anxiety, depression and suicidal thoughts in this latter population group [37, 79, 85, 91] and significant increases were found in binge eating, internet/social media usage, and gaming [87]. Data also suggests that young women are more vulnerable compared to young men [14, 89], and individuals previously diagnosed with a psychiatric disorder have an increased risk for mental-health problems [1, 56]. Moreover, the results of a large cross-sectional survey conducted in China indicated that mental health symptoms may be common especially among infected individuals [71].

There is a large body of literature analyzing different aspects of mental health and its associated factors during the COVID-19 pandemic. In Canada, Iftene and colleagues (2022) found that female gender, history of self-harm, and family conflicts were risk factors for poor mental health during the lockdown. In Russia, a study of the interactions between anxiety levels and life habit changes in the general population during the lockdown showed that decreased physical activity, sleep disturbances, and excessive internet browsing for information regarding COVID-19 were risk factors for increased levels of anxiety [74]. Moreover, during the pandemic the perception of loneliness was found to be a risk factor for psychopathological symptoms among both adolescents and adults [20]. COVID-19 related worry and rumination were associated to higher levels of anxiety and depression [2, 53, 75], as well as lower well-being and higher perception of loneliness [75]. On the other hand, resilience and enhanced social support [61], as well as keeping a basic routine during the lockdown and a good quality of sleep [36] emerged as protective factors, as they seemed to support lifestyle changes and re-adaptation mechanisms during the pandemic.

These findings highlight the importance of assessing people’s psychological condition during the pandemic and the factors associated to their mental health outcomes. However, the majority of studies were conducted during the first waves, while fewer included the fourth wave [8, 11], finding significant differences regarding the impact on mental health of the first waves compared to the last ones. Moreover, there is a lack of Italian studies specifically aimed at broadly assessing such aspects among young people.

To fill this gap, this study aimed to evaluate the mental health status among a group of Italian adolescents and young adults during the fourth wave of COVID-19 pandemic, i.e. after a persisting pandemic situation, and to identify factors associated to a good or a poor mental health status to define mental health student profiles. More in detail, our research question was the following: "Which are the main risk and protective factors associated to mental health in an Italian sample of students during the fourth wave of COVID-19 pandemic and, secondly, which mental health status profiles can be identified among such population?". Based on the available literature [1, 2, 36, 53, 56, 61, 74, 75, 87] we expect that female gender, history of diagnosed psychiatric disorder, history of self-harm and/or binge eating behaviors, sleep disturbances, anxiety and/or depressive symptoms, decreased physical activity, family conflicts, being tested positive to COVID-19, and excessive internet/social media usage were potential risk factors for mental health, while resilience, enhanced social support, and having kept a basic routine during the lockdown would appear to be protective factors. Yet, we hypothesized that some factors may arise as being the most significant and that they might contribute in defining some specific student profiles. Identifying such factors and profiles could therefore allow practitioners to recognize specific areas of intervention for promoting mental health among students at this time of the pandemic.

## Methods

### Study design and participants

The study was conducted in Brescia, a medium-sized town in Northern Italy. Together with Bergamo, another medium-sized town located near Brescia, they were the two most strongly impacted towns during the first wave of the pandemic. Fifteen Italian high schools and a medium-sized Italian university, the University of Brescia, were invited to take part in this cross-sectional observational study. Nine high schools (four scientific lyceums/grammar schools, two technical institutes and three professional institutes) and the University of Brescia agreed to participate; recruitment and data collection took place in November 2021 and lasted 2 weeks. At that time, measures taken by the government to contain the spread of the virus allowed high school students to attend their classes in person, while university students were alternating between in person and online classes online. Out of the 11,839 high school students and the 15,000 university students in the study population, 7,148 (26,6%) agreed to participate by accessing the online survey. The response rate was of 43.9% for high school students and 13% for university students. Most students were women and most of them were between 18 and 25 years old.

A multidimensional online survey was created with the Google Forms software; in order to avoid having a big amount of missing data, through the Google Forms settings we set as mandatory most questions and every item of all standardized tools. We then forwarded the survey’s access link to the representatives of each participating institute. Consequently, all enrolled students aged 14–25 received an e-mail from the student administration offices including the link to access the survey and a detailed description of the study, as well as information on their voluntary participation and on the anonymity of the collected data. Through the web link, students were asked to confirm their consent to participate. The study was conducted in accordance with the World Medical Association's Helsinki Declaration for Human Studies, and has been approved by the Ethical Review Board of the coordinating center (protocol number: 160/2021, May 28th 2021).

### Instruments

The online survey assessed several aspects of students’ mental health and well-being, including socio-demographic characteristics, anamnesis for mental disorders, and substance use. Students completed a multidimensional battery including the following measures:

- Generalized Anxiety Disorder Questionnaire (GAD-7, [77]) to measure anxiety symptoms;
- Severity Measure for Depression—Adult (adapted from Patient Health Questionnaire—9 [PHQ-9]), and Severity Measure for Depression—Child Age11–17 [4, 5] adapted from the modified version for adolescents of PHQ-9 [38] to assess depressive symptoms,
- Somatic Symptom – Adult, and Somatic Symptom – Child Age 11-17 [4, 5], both adapted from the Patient Health Questionnaire Physical Symptoms (PHQ-15, [40] to assess somatic symptom severity,
- PROMIS Emotional Distress—Anger—Short Form, and PROMIS Emotional Distress—Calibrated Anger Measure—Paediatric [4, 5] to measure the severity of anger;
- Post Traumatic Growth Inventory—short form (PTGI-SF, [16, 62], measuring the extent to which individuals, in hindsight, report positive life changes after a major life crisis,
- UCLA Loneliness Scale (UCLA, [66], designed to measure one’s subjective feelings of loneliness as well as feelings of social isolation,
- Connor-Davidson Resilience Scale 10 items (CD-RISC-10, [15] describing different aspects of resilience,
- Fear of COVID-19 Scale, Italian version [75], a seven-item scale that assesses the fear of COVID-19,
- Sleep Problems Domain of the DSM-5 Self-Rated Level 1 Cross-Cutting Symptom Measure—Adult, and DSM-5 Self-Rated Level 1 Cross-Cutting Symptom Measure—Child Age 11-17 [4, 5] to measure the severity of sleeping problems;
- A selection of items included in an adapted version of the Risky Behavior Questionnaire for Adolescents (RBQ-A, [7]), a 20-item self-report instrument which assesses broad-based engagement in risky behaviors in the past month.

More details on scales and questionnaires are reported in Additional file 1: List S1. We also included a selection of questions regarding the activities in which students had been engaged during lockdown (i.e. hobbies, sports, time spent online, having met friends online), and questions regarding their family relationships. More specifically, we asked them to rate their satisfaction as to the quality of their family relationships on a scale from 0 = *not at all satisfied* to 10 = *extremely satisfied.* Finally, we assessed the occurrence of a number of COVID-19 related events, such as death or hospitalization for COVID-19 of a loved one, and COVID-19 test positivity of the students themselves or of a loved one during the pandemic.

### Data analysis

Descriptive statistics for socio-demographic, academic characteristics and for the questionnaire scores were computed by percentage distribution for categorical variables, and mean and Standard Deviation (*SD*) for quantitative variables. Association analyses between gender and the outcomes of instruments were examined using the Mann–Whitney test.

In order to highlight any potential student subgroups in terms of the clinical scales, an unsupervised hierarchical clustering analysis was performed. The four scales used as a “proxy” for mental health status were PHQ-9, GAD-7, PROMIS and PHQ-15. Hierarchical clustering is a data-driven approach that assigns the subjects to different clusters, which number depends on the similarity of the observations on the four clinical scales. The outcome of this method is a dendrogram where the resulting clusters are highlighted [50]. We verified that this classification was consistent from a clinical point of view by calculating the mean scores of the four scales for each cluster.

At this point, K-means clustering was implemented. This technique consists in a different approach to clustering, in which the number of clusters is decided a priori. Subjects are allocated to a given cluster following a distance criterion: each subject is assigned to the cluster with the closer centroid to the subject itself [82]. The chosen number of clusters was the one resulting from the hierarchical clustering. Data was standardized before clustering.

We assessed the robustness of this classification by calculating the accuracy (i.e., the accordance between the number of subjects with most severe mental health problems according to the K-means method, and the number of subjects with most severe mental health problems according to the cutoffs of the standardized clinical scales). We calculated the accuracy for the four clinical scales by evaluating the four confusion matrices between the categories of mental health status defined by the cutoffs and the categories of mental health status defined by K-means clustering.

After that, we identified (in line with previous findings including [20, 36, 56, 61, 74, 89] a pool of variables potentially associated to the two mental health groups defined by clustering analyses and we used a random forest model in order to assess which of these variables were most significantly associated to the categorical variable mental health status (coded 0 for good mental health and 1 for poor mental health, according to cluster assignation). Random forest is an ensemble method of machine learning, very useful to perform feature selection, ranking a pool of variables according to their importance in discriminating between the categories of the dependent variable [69]. The output of the random forest, which consists in a double grill, ranks variables according to two different measures, namely the Mean Decrease Accuracy (MDA) and the Mean Decrease Gini (MDG). The most predictive variables were selected by taking into account both indexes. The accuracy score was calculated for the random forest model.

Univariate and multivariable logistic regressions were performed to confer robustness to the random forest’s results. In univariate models the individual effect of each variable on the dichotomized mental health status was quantified by computing odds ratio and their predictive power was evaluated with AICs. Then, only the most predictive variables resulted from the random forest were tested together for association with mental health status in a multivariable model.

To identify the mental health student profiles, a classification tree (with mental health status categories as labels) was run on the most predictive variables selected by previous method. The output of this model consists in a series of classification paths identified by the cutoff values of the regressors. Each node of the tree contains a specific percentage of subjects, which are allotted to a class and a probability of class assignment. The accuracy score was computed for the classification tree model. All tests were two-tailed and the probability of a type I error was set at p < 0.05. The analyses were performed using R software (R Core Team, 2021, version 4.1.0, package “cluster”), except for the classification tree analysis, for which SPSS software (v. 28) was used.

## Results

### Descriptive statistics and association analyses

Out of the 26,839 students in the initial study population, 7,229 accessed the online survey and 7,148 (26,6%) agreed to participate. Following data quality control, we discarded from the analysis two students who seemed to have provided random answers (e.g. they selected the first option for each question and for each item of the standardized scale), resulting in a final sample of 7,146 students. All students in the final sample completed the survey, with just one student not providing any information on their current housing situation and 21 of them not indicating the number of people they live with. Table 1 shows the characteristics of the sample and the prevalence of COVID-19 related events in the study group.

**Table 1:** Sample characteristics and COVID-19 related events

	n	%
Gender		
Female	4507	63.1
Male	2533	35.4
Other	106	1.5
Age range		
14–15	2090	29.3
16–17	1903	26.6
18–25	3153	44.1
Education		
Scientific Lyceum and Grammar Schools	2432	34.1
Technical institutes	2045	28.6
Professional institutes	724	10.1
University	1945	27.2
	Yes (%)	No (%)
Diagnosis of any mental disorder (lifetime)	738 (10.33)	640 (89.67)
COVID-19 related events		
Someone dear to me has died from COVID-19	1391 (19.5)	5755 (80.5)
I tested positive for COVID-19	1017 (14.2)	6128 (85.8)
Some people dear to me have tested positive for COVID-19	5276 (73.8)	1870 (26.2)
Some people dear to me have been hospitalized for COVID-19	2222 (31.1)	4924 (68.9)
I have not had any of the previous experiences with COVID-19	1684 (23.6)	5462 (76.4)

Most students had a close person who had tested positive for COVID-19, while less than one in five had a close one who died from COVID-19. The great majority of students lived with their parents (96%), and they were quite satisfied with the quality of their family relationships (mean score = 7.32, SD = 2.04). During lockdown, one in four students (24.7%) spent more than five hours online, while 40% of the sample often played sports. More than half of the sample frequently met with their friends online (56%), while 13% engaged in volunteering activities. About 66% of the participants reported to have continued to pursue their habitual hobbies, while 54% found new activities to practice, at least sometimes. Table 2 shows the total mean scores for all instruments and the prevalence of sleep problems and risky behaviors, by gender. The rate of students scoring above the cutoff is given for PHQ-9, PHQ-15, GAD-7 and PROMIS anger, as for these tools it was possible to collect robust studies providing cutoff score indications [4, 5, 60]. Additional file 1: Table S1 shows the frequencies of each risky behavior in our sample (see Additional file 1).

**Table 2:** Standardized tools’ scores and risky behaviors by gender

	Total		Males		Females		Test statistic	*p*^a^	Effect size (CI 95%)
	Mean (SD)	Above cutoff	Mean (SD)	Above cutoff	Mean (SD)	Above cutoff			
*Standardized tools*									
PHQ-9	9.43 (6.51)	42.8%	6.93 (5.62)	27.3%	10.72 (6.49)	51.0%	*U*st = −24.70	< 0.001	0.35^b^ (0.33–0.38)
GAD-7	9.54 (5.86)	41.8%	6.94 (5.28)	27.6%	10.96 (5.63)	57.3%	*U*st = −28.26	< 0.001	0.41^b^ (0.38–0.43)
PHQ-15	8.15 (5.85)	34.6%	5.33 (4.63)	14.8%	9.66 (5.83)	45.1%	*U*st = −31.79	< 0.001	0.46^b^ (0.43–0.48)
PROMIS (T-score)	52.50 (12.57)	46.8%	48.32 (12.02)	28.0%	54.76 (12.21)	49.3%	*U*st = −20.93	< 0.001	0.30^b^ (0.27–0.33)
Fear of COVID-19	14.80 (5.04)		13.14 (4.59)	–	15.75 (5.03)	–	*U*st = −21.51	< 0.001	0.31^b^ (0.28–.33)
UCLA	20.69 (13.93)		17.42 (13.26)	–	22.32 (13.89)	–	*U*st = −14.92	< 0.001	0.21^b^ (0.19–0.24)
CD-RISC-10	17.79 (8.28)		20.12 (8.15)	–	16.57 (8.00)	–	*U*st = 17.48	< 0.001	0.25^b^ (0.22–0.28)
PTGI-SF	20.95 (10.68)		18.49 (10.79)	–	22.49 (10.25)	–	*U*st = −14.92	< 0.001	0.21^b^ (0.19–0.24)
	Yes (%)	No (%)	Yes (%)	No (%)	Yes (%)	No (%)		*P*^c^	
*DSM5 Cross-Cutting -Sleep problemsSymptoms*	32.3	67.7	22.2	77.8	37.6	62.4	χ^2^ = 176.03	< 0.001	0.16^d^ (0.14–1)
*Risky behaviors*^*e*^	Yes (%)	No (%)	Yes (%)	No (%)	Yes (%)	No (%)		*P*^c^	
Self-harm	28.4	71.4	22.8	77.2	31.9	68.1	χ^2^ = 64.57	< 0.001	0.10^d^ (0.08–1)
Binge eating	37.3	62.7	30.4	69.6	41.3	58.7	χ^2^ = 81.83	< 0.001	0.11^d^ (0.09–1)
Reckless driving	20.9	79.1	32.5	67.5	14.4	85.6	χ^2^ = 319.68	< 0.001	0.21^d^ (0.19–1)
Binge drinking	42.2	57.8	44.0	56.0	41.2	58.8	χ^2^ = 5.29	0.21	0.03^d^ (0–1)
Cannabis use	7.5	92.5	10.1	89.9	6.1	93.9	χ^2^ = 38.33	< 0.001	0.07^d^ (0.05–1)

*PHQ-9* Severity Measure for Depression adapted from Patient Health Questionnaire- 9, *GAD-7* Generalized Anxiety Disorder Questionnaire, *PHQ-15* Somatic Symptom adapted from the Patient Health Questionnaire Physical Symptoms, *PROMIS* PROMIS Emotional Distress – Anger, *UCLA* UCLA Loneliness Scale, *CD-RISC-10* Connor-Davidson Resilience Scale, *PTGI-SF* Post Traumatic Growth Inventory -short form, *t* statistic *t* of the *t*-test, *U*st standardized Mann–Whitney *U* statistic, *CI* Confidence Interval. The cutoff score used for PHQ-9, GHQ-7 and PHQ-15 was 10, and the cutoff score used for PROMIS was 55 (T-score)

^a^Mann-Whitney test

^b^Cliff’s delta; d < 0.147: negligible effect, 0.147 < d < 0.330: small effect, 0.330 < d < 0.474: medium effect, d > 0.474: large effect [64]

^c^Chi squared test

^d^Cramer’s V; 0.1: small effect, 0.3: medium effect, 0.5: large effect [23]

^e^*Yes* corresponds to Mild, Moderate or Severe for Sleep Problems. *Yes* corresponds to 1 = Almost never, 2 = Sometimes, 3 = Quite often, and 4 = Often for Self-harm, Binge eating, Reckless driving and binge drinking; *Yes* corresponds to 2 = Sometimes, 3 = Quite often, and 4 = Often for cannabis use

Focusing on the gender differences, female students scored significantly higher than males in all scales except for the CD-RISC-10 (see Table 2).

Referring to the rates of risky behaviors, measured with an adapted selection of RBQ-A items, results indicated that more than 40% of students engaged in binge drinking (i.e. binge drinking episode and/or drinking with the purpose of getting intoxicated) at least once in the last month, while more than one in three reported binge eating behaviors (i.e. having purged or binged). A significantly higher percentage of male students, compared to female students, used cannabis and engaged in unsafe driving (i.e. driving a bicycle, a moped, and/or a car at high speed, or under the influence of a substance etc.), while more female students reported binge eating and self-harming behaviors (i.e. intentionally injuring or inflicting pain on their body, for example through cuts or burns, without suicidal intentions) in the past month compared to their male counterparts (Table 2). However, it should be noted that the effect sizes for all the comparisons of risky behavior frequencies between males and females were small. The Sleep Problems Domain of the DSM-5 Self-Rated Level 1 Cross-Cutting Symptom Measure, used to assess sleep problems, evaluated sleep problems through a single question which asked how often, in the last 2 weeks, the person had been bothered by sleeping problems that affected their sleep quality over all. Again, female students reported higher levels of sleep problems compared to male students.

In Mann–Whitney tests, as well as in the subsequent algorithms and classification models, we discarded the gender category "Other" because the subsample size was very small compared to the other categories (namely, "Male" and "Female"). However, we performed descriptive statistics for such subsample, and results revealed high mean scores in PHQ-9, PHQ-15, GAD-7, UCLA, and low mean scores in PTGI-SF and CD-RISC-10. Half of students who identify themselves in the gender category "Other" showed risky behaviors in terms of self-harm and binge eating, and many of them (from 15 to 41%) reported cannabis use, binge drinking, and reckless driving (Additional file 1: Table S2).

### Cluster analyses

**Fig. 1:**
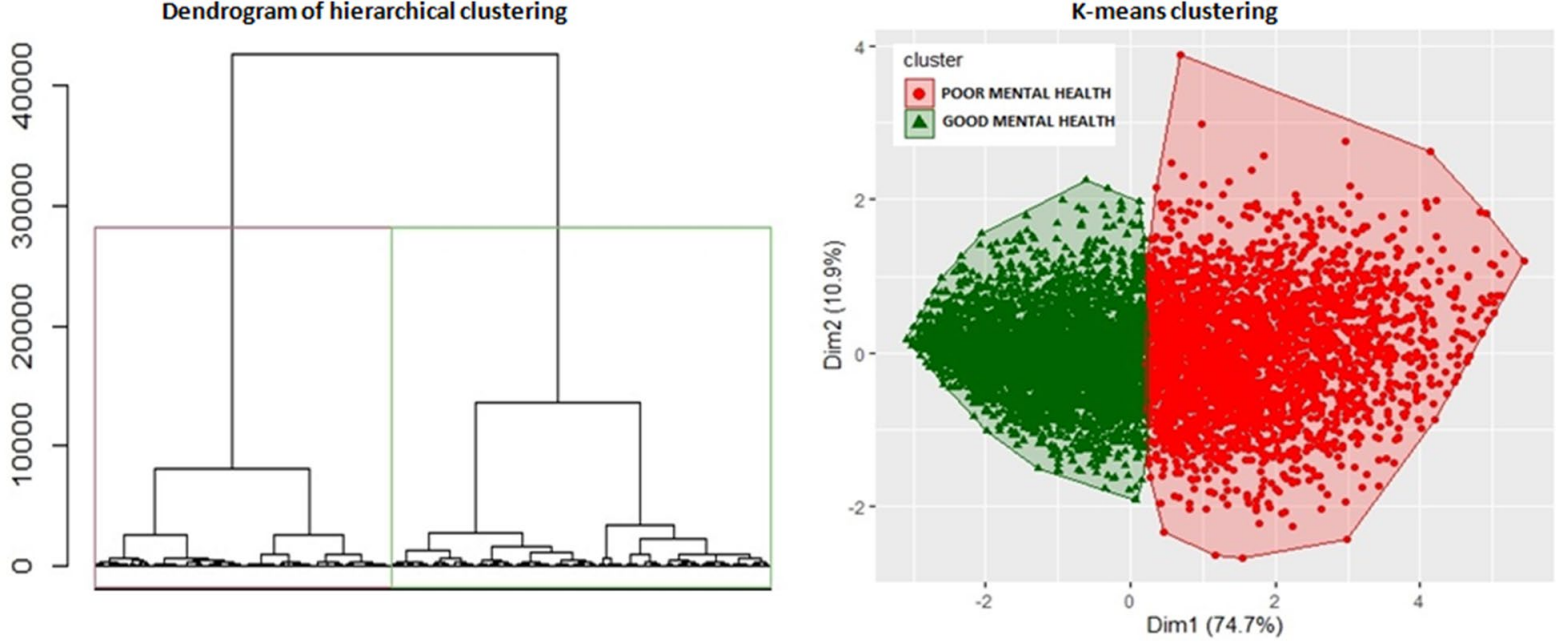
Dendrogram of hierarchical clustering and k-means clustering

Aiming to identifying student subgroups in terms of the clinical scales, we performed a hierarchical clustering procedure, which outputted a dendrogram displaying two clearly separate clusters. Mean scores of all four scales were calculated for each cluster, to assess if the procedure was accurate from a clinical point of view (Additional file 1: Table S3A). The mean scores for all scales were clearly lower compared to the other: the two groups structure, detected by the clustering procedure, has found a clear clinical correspondence. Based on this evidence, the K-means clustering procedure was performed, imposing a number of clusters equal to two (Fig. 1).

Students appeared to be perfectly separated in two distinct groups: the red cluster includes students with poor mental health (n = 2,999), while the green cluster includes students with good mental health (n = 4,040). The confusion matrix of the K-means clustering classification of mental health groups with respect to the clinical scales cutoff classification of mental health groups showed accuracy levels of 86.3% for PHQ-9, 85.4% for GAD-7, 83.8% for PROMIS and 82% for PHQ-15 (Additional file 1: Figure S1 and Table S3B). The accuracy for all scales was high enough to claim that the clustering classification holds from a clinical point of view as well. The silhouette analysis further confirmed that the optimal number of clusters for this data was two (Additional file 1: Figure S1).

### Random forest and classification tree analyses

In order to identify the variables associated to mental health status, we labelled all subjects with their assigned cluster by creating a dichotomous variable (coded 0 for "good mental health" and 1 for "poor mental health"), and tested 22 potential risk and protective factors that we included in the data collection to associate to such variables. These factors included socio-demographic, clinical and behavioral characteristics, as well as COVID-19 related events, in line with previous findings and listed in Additional file 1: Table S4.

First, the most significant variables were selected by using the random forest procedure (Additional file 1: Figure S2). MDA (Mean Decrease Accuracy) and MDG (Mean Decrease Gini) scores for all variables were calculated and both these scores were taken into account to identify seven highly discriminating variables (i.e. the ones most significantly associated to the dichotomous variable mental health). The identified variables were: UCLA, Self-harm behaviors, CD-RISC- 10, Satisfaction with family relationships, Fear of COVID-19 Scale, Gender and Binge eating behaviors.

### Logistic regression models

Univariate logistic regressions allowed to quantify the effect of each variable on dichotomized mental health using odds ratios, as well as Akaike Information Criterion (AIC; the best discriminating variables were the ones appearing in the univariate models displaying the lowest AICs). Results showed that the seven most discriminating variables resulting from random forest were also the most discriminating variables in logistic univariate models. Among the other variables, age (minor vs. adult), education, lifetime diagnosis of mental disorder, and daily time spent online were also significant. The multivariable model was performed to further confirm the results of random forest (Additional file 1: Table S4). Indeed, some of the best variables resulting from random forest could have appeared at the top of the grills because they were actually correlated to other discriminating variables. However, all seven variables were highly significant in the multivariable model as well, conferring robustness to the random forest results.

### Classification tree analysis

**Fig. 2:**
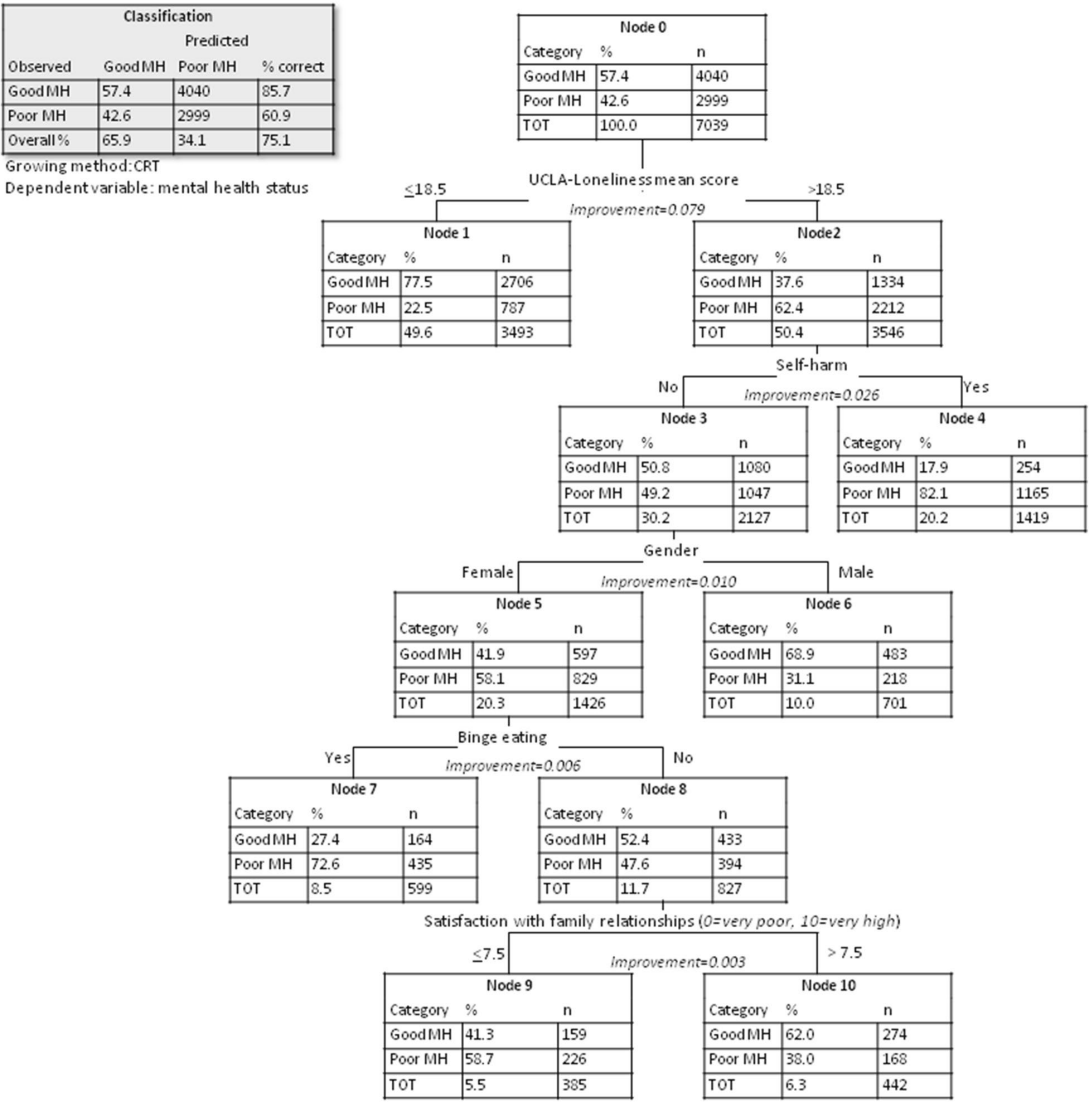
Classification tree

Classification tree analysis (Fig. 2) allowed to use the most discriminating variables emerged from the random forest to define the students’ clinical profiles. The tree was able to display five of the seven most important variables identified with the previous model. Globally, poor mental health was well defined by higher scores on UCLA and the presence of self-harm behaviors: 82.1% of students with higher loneliness (with UCLA score > 18.5) and with self-harm behaviors (n = 1,165) were in the "poor mental health" category. Instead, good mental health was well defined by lower levels of loneliness: 77.5% of students with UCLA score < 18.5 (n = 2,706) were in the “good mental health” category.

Moreover, among those students feeling lonelier, but without self-harm behaviors, 68.9% of male students were in the good mental health category (n = 483), compared to only 41.9% of their female counterparts (n = 597). Among the female subgroup, those with binge eating behaviors had 72.6% chance of belonging to the "poor mental health" category (n = 435). On the other hand, when considering the same subgroup students without binge eating behaviors, the variable that pointed towards better mental health appeared to be the satisfaction towards family relationships (62% of cases, n = 274).

All the analyses were repeated considering the risky behaviors as polytomous variables with three categories (0 = Never, 1 = Almost never, 2 = Sometimes, Quite often or Often). We obtained the same results as the previous analysis, therefore we can claim the results to be robust (see Additional file 1: Figure S3A and B).

## Discussion

The present study drew a clearer picture of the mental health condition of a large group of Italian students during the fourth wave of the COVID-19 pandemic, confirming that the latter caused major psychological distress and disruptions to everyday life. The impact of such disruption on mental health is of critical policy concern, and, among those identified as a specific population of concern, were adolescents and young adults. Notably, due to the nature of the study, it is not possible to clearly identify the overall burden of COVID-19 pandemic on mental health condition, however the present results showed high level of depressive, anxiety, somatic and anger-related symptoms and several potential risk-factors associated to poor mental health that should be addressed. Our study can contribute to identifying potentially at high-risk adolescents and young adults that could be the target for specific preventative actions.

The present study aimed to evaluate the mental health status of a large group of Italian adolescents and young adults during the fourth wave of COVID-19 pandemic, and to identify potential risk and protective factors and student profiles associated to their mental health status. Nearly 27% of the students of our sample completed the web-survey, with a significant difference in the response rate between high school students and university students. This difference relied on the fact that, in high schools, the staff usually has a closer relationship with students and therefore has more opportunities to engage students in the survey. University, on the other side, could only solely on emails as a mean to promote the survey.

### Clinical characteristics and risky behaviors

In our study, more than 40% of students showed moderate or severe depressive and anxiety symptoms. Such rates are higher than those presented in several studies on young people during pandemic [33, 58, 91], but quite similar to others [44, 80]. When compared to pre-COVID Italian data, our results still show slightly higher rates of depressive and anxiety symptoms compared to both adolescent and college student samples [17, 55]. Additionally, significant somatic and anger-related symptoms were significantly very high compared to other international studies [45, 63], but quite similar to the results from another Italian study on college students which evaluated somatic symptoms before and during the pandemic [17]. This variability could be explained considering different factors, such as the sample’s characteristics (e.g. different age ranges, as for [58, 63] which included adults in their samples), cultural gaps (e.g. Asian cultures described by [91] and [80], which greatly differ from Italian culture), and differences in the way countries were affected and dealt with COVID-19 pandemic throughout time [45, 58] Liu et al. were conducted in the first wave of the pandemic). The fact that Italy was the first European country to be severely impacted by the pandemic, and that Brescia specifically was one of the most affected cities in the country, may have had a major impact on people’s mental health, especially the youngest, and this could explain such high rates. This study, therefore, might also be considered as a picture of the detrimental impact of the pandemic on young people living in Brescia, and it may help stakeholders and healthcare providers tailor specific and local mental health interventions.

UCLA scores were quite low, indicating that the students did not experience intense feelings of loneliness and social isolation, probably due to the time point of the survey. Indeed, it should be noted that the present survey was conducted during the fourth wave of the pandemic: restrictions had been loosened and schools had been reopened, allowing students to meet their peers and engage in social interactions. Fear of COVID-19 scale scores were also quite low, indicating that the students of our sample were not excessively fearful of COVID-19. Again, during the fourth wave of the pandemic, a large amount of research contributed to understand that the variants of SARS-CoV-2 spreading during the fourth wave caused symptoms of lower severity compared to the first waves, and vaccination campaigns had been implemented. These factors may have had a relevant impact on people’s fear of COVID-19 at that specific timepoint.

Among potential protective factors, we found that resilience was quite low among students, but it was in line with results found from other European studies [25, 30]. It is also worth noting that, in this study, students reported, in hindsight, only few positive life changes following a potentially traumatic event; Wu and colleagues [88] considered 60% of the highest PTGI-SF score (therefore, a score of 30) as the standard for medium and above levels for post traumatic growth, while in our sample the mean score was around 18. This could be generally in line with previous Italian studies conducted before the pandemic: for example, Bianchini and colleagues [12] conducted a study on college students after the earthquake in L'Aquila (an Italian city in the Abruzzo region) in 2009, finding a PTGI (not the short form of the scale) mean score of 35.23 (SD = 21.1), which indicated a low level of post-traumatic growth (as they considered a score equal or above 57 as the standard for medium and above level of post-traumatic growth). Further research is needed to shed light on this quite unexpected result, but we hypothesize that such low levels might be influenced by the long duration and the wave pattern of the pandemic, and the fact that more than 2 years have gone by since the COVID-19 outbreak. Sleep problems affected a third of our sample; this is in line with other studies on the negative impact of COVID-19 on Italian adolescents’ sleep during different national lockdown periods [3, 9] and during spring in 2021, a later phase of the pandemic that included less severe confinement measures [9].

Regarding risky behaviors, in our sample the prevalence was very high (with an exception for cannabis use, observed in 7.5% of the sample), with male students using cannabis and engaging in unsafe driving significantly more often that female students. On the other hand, females were more prone to binge eating and self-harming behaviors. Binge drinking was very common in both genders, although, statistically, a significant higher percentage of males engaged in such behaviors. Again, such gender differences are in line with previous research [13, 42, 59, 76]. However, it should be noted that the effect sizes of all comparisons were small (Cramer’s V < 0.3), ranging from 0.03 (binge drinking) to a maximum of 0.21 (reckless driving). Therefore, the magnitude of such differences and the implications for practice are limited.

In addition to the gender differences cited above, female students scored significantly higher than male students on all scales except for the CD-RISC-10, indicating higher distress and lower resilience, and this matched with other findings reported in literature [21, 57, 84]. In line with previous research that considered this difference to be based on a tendency, for women, to use more positive appraisals as coping strategies to deal with traumatic or negative events [52, 88], in our study female students also reported slightly higher levels of post-traumatic growth than males. Such indicators of psychological distress in female students may result in future problems in functioning, health and psychological well-being (e.g. onset of common mental health disorders such as depression and anxiety). Adolescence is generally when the gender gap in mental health emerges, and this may play a role in the disproportionately higher prevalence of mental health disorders in women worldwide [39]. Although such difference remains poorly understood, many of the explanations include a combination of biological and social factors [14, 85]. A more in-depth understanding of the factors associated to gender differences in young people's psychological well-being is therefore fundamental, and future studies are needed to address such unanswered questions. It should be noted that despite the high significance of the tests, the effect sizes of these comparisons were rather small, except for the male–female comparisons of the PHQ-9, PHQ-15 and GAD-7 scales, for which medium effect sizes have been found. Therefore, as for risky behaviors, the magnitude of the gender difference regarding loneliness, anger, resilience, fear of COVID-19 and post-traumatic growth should be considered limited, while it confirmed its clinical relevance for depressive, somatic and anxiety symptoms.

### Identification of subgroups in terms of mental health, evaluation of factors associated to good or poor mental health status and definition of students’ profiles

The hierarchical clustering procedure identified two clearly separate clusters and this was validated from a clinical point of view by calculating the mean scores of the four scales by cluster. All mean scores were lower in a cluster and higher in the other. According to this evidence, K-means clustering was performed, imposing a number of clusters equal to two, reflecting groups of adolescents and young adults with different psychological features that we defined as "poor mental health" and "good mental health". This classification was consistent from a clinical point of view, considering the accuracy being high for all four selected clinical scales, and gave us the chance to study the associated students' profiles and to detect the potential risk or protective factors for mental health status by using the random forest method. This analysis showed that the variables that best discriminated between "poor mental health" and "good mental health" were: loneliness (UCLA score), self-harm behaviors, resilience (CD-RISC-10 score), satisfaction with family relationships, fear of COVID-19 (Fear of COVID-19 Scale score), gender, and binge eating behaviors. It is very interesting to note that loneliness was such a strong discriminating variable, and the results of a recent systematic review [20] may support our findings, as they found loneliness to be related to depressive symptoms, generalized anxiety and with post-traumatic stress, and also contributing to the persistence of other psychopathological symptoms. Interestingly, in our sample the UCLA scores were not particularly high, but its effect on mental health status seems to be strong. Furthermore, the link between the occurrence of risky behaviors such as self-harm and binge eating, and poor mental health is well documented [41, 86], as well as for gender differences [21, 57, 84]. A recent longitudinal study [55] including a pre-post pandemic evaluation found that after controlling for baseline mental health status, those adolescents reporting an increase in self-harm, binge-drinking, aggressiveness, and binge-eating were more likely to present a worsened mental health status. Moreover, resilience well discriminated between poor and good mental health clusters and this may be read in light of those studies that found this variable to be a protective skill for mental health [30, 34, 54]. Fear of COVID-19 has been previously associated to depression and anxiety [2, 75], and this is in line with our results confirming the Fear of COVID-19 Scale score to be one of the variables most significantly associated to mental health. Finally, the quality of family relationships was well discriminating among mental health clusters. Such result is in line with a recent review describing family relationships as a factor associated to child and adolescent mental health during pandemic [35].

The logistic regression analysis confirmed that the seven variables selected with random forest were the most discriminating between good and poor mental health. Foreseeably, among results there were other interesting associations, such as between current mental health status and a lifetime diagnosis of a mental disorder. Although this variable was not one of those best discriminating among mental health clusters, it is well documented how the current COVID-19 pandemic and related restrictions are psychosocial adversities that may cause an exacerbation of symptoms among people with a prior mental health diagnosis [1, 56, 83]. Another variable that increased the chance of being in the category of "poor mental health" was spending five or more hours online. Spending an excessive amount of time online and excessive media consumption were found to be associated to depressive and anxiety-related symptoms, and negatively associated to overall well-being [18, 51]. The univariate logistic regressions also showed that attending university increased the possibilities of being in the category "good mental health" when compared to scientific lyceum and grammar schools, and with technical institutes. This may be in line with the fact that being adult (+ 18 years old) was also found to be associated to an increased chance of belonging to the category "good mental health", and university students are all over 18. It is possible to hypothesize that, although still young and in a critical period of transition from childhood to adulthood, university students had more opportunities to gain more skills and to access more resources over time in order to cope better with mental health difficulties, compared to high school students. Lastly, COVID-19 test positivity was not associated to poor or good mental health, differently from what [71] suggested,nonetheless, considering the events related to COVID-19 experienced by the students of our sample, results suggest the importance of developing specific interventions for those who have lost or risked losing a loved one(s), as the trauma suffered was significantly associated to poor mental health.

The classification tree analysis permitted to define students’ profiles based on the most discriminating variables defined by the random forest. Loneliness and self-harm behaviors were the most predictive variables for poor mental health: poor mental health status was defined by higher scores of UCLA (a score of 18.5 emerged as a cutoff), while more than three out of four students scoring below such cutoff were in the good mental health category. Nevertheless, amongst students reporting feelings of loneliness, 68.9% of males displayed good mental health, while 58.1% of females displayed poor mental health. This result suggests that loneliness has a greater negative impact on women's mental health than on men's, and further studies are needed to better understand such difference. Moreover, the classification tree showed that 82.1% of students with UCLA score > 18.5 and with self-harm behaviors in the last month fell in the "poor mental health" category. Such results are not surprising: the association between loneliness and self-harm behaviors in adolescence has increasingly drawn researchers’ attention [65]. Our findings are in line with a recent cross-sectional school survey reporting that the exacerbation of loneliness during the lockdown period was associated to an increase in the odds of self-harming in the same period among UK adolescents [26] another study [78] found that COVID-19-related loneliness (assessed in March 2020) predicted higher depressive symptoms for all adolescents, higher non suicidal self-injury frequency for adolescents with low pre-pandemic frequency (but less frequent non suicidal self-injury for adolescents with high pre-pandemic frequency), and higher suicide risk for adolescents with higher pre-pandemic risk. Lonely females, without self-harm behaviors, but presenting binge eating behaviors were classified within 72.6% of cases as subjects with poor mental health. This result confirmed once again the link between such behavior and negative mental health outcomes. Lastly, satisfaction regarding family relationship emerged also in this analysis as a predictive variable for good mental health, but only among female students reporting feeling of loneliness and without self-harm or binge eating behaviors.

### Implications for school-based prevention programs

Results from this current study highlighted that adolescents and young adults having experienced previous mental health problems were at risk for poorer mental health after the COVID-19 pandemic. However, at the same time, students not previously identified as at risk were found to face mental health challenges. Preventative programs directed to the early warning signals of psychopathology such as a depressed mood, increased anxiety or anger could be effective targets for the prevention of mental health consequences in the post-pandemic period among adolescents and young adults. Preventative efforts on developing social skills programs are needed in order to reduce experiences of loneliness and the mental health burden experienced by students [49]. Upstream prevention interventions at a universal level that are directed at reducing depression or emotional dysregulation could also show potential in decreasing at risk behaviors as a ripple effect. Recently, social-emotional learning programmes for adolescents based on cognitive-behavioral therapy and mindfulness practices [10, 29], and dialectical behavioral therapy [24, 47, 90] showed the feasibility and preliminary efficacy in different school settings. Considering that among protective measures to address factors associated with good mental health, the positive association between physical activity and other health education habits (e.g. nutrition) with well-being, resilience and emotional functioning among adolescents from the general population has been largely documented [48, 68], multi-component interventions focused on mental health literacy and physical activities could be potentially useful in increasing exercise and physical activity frequency while promoting psychological well-being and self-care [31]. Furthermore, targeting social factors such as social isolation and family climate along with engagement, from both school administrators and teachers, are essential for the implementation of school prevention programs.

### Strengths and limitations

This study has strengths and limitations. The main strength is the inclusion of multidimensional assessment that allowed to evaluate mental health status taking into account a broad number of variables and to identify potential risk and protective factors. Secondly, the large sample allowed us to apply the unsupervised data-driven clustering approach in a robust and reliable way, enabling to highlight a clear clinical classification structure of data. The sample size was very large, different types and degrees of education were included, and the range of students’ age (14–25) was wide; however, it should be noted that our participants may not represent the best sample for generalizing results. Indeed, it was not representative on a national scale as it came from a single city in Northern Italy. Collaborations in the future with other institutions or researchers from different regions or countries could provide more representative data from multi-site studies. Moreover, as participation was voluntary, students who chose to participate may differ from those who declined the invitation and it was not possible to collect any information from the latter. Globally, the response rate was good although it was relatively low among university students, which again raises concerns about the representativeness of the sample; unfortunately, we could not consider providing incentives to students to participate, in order to increase the response rate. Available data on web-surveys among students’ populations, though, show a variable response rate, ranging from 10% to more than 90% [6, 19, 32, 43, 72] and, as suggested by previous research [22, 67], the odds of response to a web-survey can greatly vary considering the different design and characteristics of the web-survey itself. Further studies including more representative samples should therefore improve the generalizability of results.

The self-report nature of our measures can be considered as a second limitation in terms of the validity of results, as it could lead to social desirability bias or inaccurate reporting. However, this allowed the collection of a wide range of data from a large sample of students, which would not have been possible with interviews or other similar methodologies. Nonetheless, clinician-rated measures or physiological assessments might be included in future studies in order to obtain more valid data.

As a third limitation, while the study included a comprehensive battery of measures to assess various aspects of mental health and well-being, we could not include some potentially relevant constructs, such as self-esteem or coping strategies. This might have lead to the omission of some potentially relevant factors, essential for a comprehensive understanding of the mental health status of students; for example, our findings highlighted an interesting gender difference, but our measures did not allow an in-depth analysis of the factors that may be driving these differences. We decided to limit the number of questions and measures in order to avoid lower response rate often caused by longer web-surveys [22]. Further studies including a wider range of variables are therefore needed.

## Conclusions

The results of this study drew a clearer picture of the well-being of Italian students during the fourth wave of the COVID-19 pandemic and provided insights regarding the factors associated to good or poor mental health condition. Although it is not possible to consider this survey as a diagnostic assessment, our findings showed high levels of psychopathology; interventions aimed at reducing depression and anxiety and improving emotional regulation, alongside with psychosocial interventions are needed in order to reduce loneliness in light of its negative impact on good mental health. Our findings also show the need for protective measures to address when implementing prevention and promotion interventions for young people, by enhancing those aspects that are found to be associated to a good mental health. Mental health strategies aiming to improve mental health conditions of young people should therefore integrate programs targeting the enhancement of family environment and social networks. Such programs should stem from a close cooperation between policy makers, health professionals, social and educational services (including schools and university). A multi-tiered model including a continuum of interventions with different levels of support represents an innovative approach towards collaborative care in schools [46]. Gijzen and colleagues [28] planned a multimodal stepped-prevention program for depression and suicidal behaviors in adolescents that entails in the first phase an early screening and detection of suicidal behaviors and depressive symptoms, a safety net including gatekeepers at school, and in the second phase the implementation of both universal and indicated prevention programs. Stepped intervention could include referrals to mental health services when needed [73]. Moreover, policy makers could sustain informative campaigns to improve mental health literacy (especially for parents) and support social and educational services at a local level and national level. Prevention and early intervention for mental health burdens in young people are priorities. Universal school-based programs should be delivered early on, preferably starting during middle school years (ages 11–13) and the first years of high school. This would allow early identification of potential clinical antecedents such as depressive symptoms, anxiety, and anger and reduce the negative effects of exposure to social stressors (e.g. stressful life events, discordant relationships among peers or family members, social isolation).

In conclusion, although the psychological distress that the students in our sample and, more generally, young people have shown during pandemic is of critical concern, it cannot be read only as an effect of the pandemic experience and, therefore, limited to the most critical periods of COVID-19 breakouts. Timely actions on young people are strongly needed, and should consider both the long-term effects of COVID-19 on mental health status, and how it is also part of the challenges that, during the transition from childhood to adulthood, young people have to face regardless.

## Supplementary Information

**Additional file 1: List S1.** List of standardized scales. A list of the standardized scales included in the assessment. **Table S1.** Frequencies of risky behaviors, in the last month. **Table S2.** Standardized tools and risky behaviors for gender variable, ‘Other’ category. **Table S3.** A and B Mean scores of the four clinical scalesby mental health group, where groups are identified by hierarchical clustering, and confusion matrix between mental health categories identified by the four clinical scales' cutoffs and mental health categories identified by k-means clustering on the four clinical scales. **Figure S1.** Silhouette analysis displaying the optimal number of clusters. **Figure S2.** Output of random forest model: two grills displaying all variables tested for association with mental health status. **Table S4.** Results of univariate and multivariable regression models. The variables displaying the lowest AIC in the univariate logistic regressions are the seven most discriminating variables identified previously by random forest. **Figure S3**. A and B. Output of random forest model and Classification tree obtained using the polytomous version of the “risky behavior” variable.

## Data Availability

The datasets generated during and/or analysed during the current study are available in the Zenodo repository, https://doi.org/10.5281/zenodo.7599238.

## Abbreviations

AIC: Akaike information criterion
APA: American psychiatric association
CD-RISC-10: Connor-davidson resilience scale 10 items
CI: Confidence interval
COVID-19: Coronavirus disease 19
GAD-7: Generalized anxiety disorder questionnaire
MDA: Mean decrease accuracy
MDG: Mean decrease gini
PHQ-9: Patient health questionnaire—9
PTGI-SF: Post traumatic growth inventory -short form
RBQ-A: Risky behavior questionnaire for adolescents
SD: Standard deviation
UCLA: UCLA loneliness scale
Ust: Standardized Mann–Whitney U statistic
