# Supplementary material for: Risk and protective factors associated with mental health status in an Italian sample of students during the fourth wave of COVID-19 pandemic
Source: Child Adolesc Psychiatry Ment Health. 2023 Jun 26;17:78. doi: 10.1186/s13034-023-00615-w (PMC10294442; doi:10.1186/s13034-023-00615-w)

**Additional File 1**

This additional file includes:

- **List S1. List of standardized scales.** A list of the standardized scales included in the assessment.
- **Table S1.** Frequencies of risky behaviors, in the last month.
- **Table S2**. Standardized tools and risky behaviors for gender variable, ‘Other’ category.
- **Table S3A** and **Table S3B**. Mean scores of the four clinical scales (PHQ-9, GAD-7, PROMIS, PHQ-15) by mental health group, where groups are identified by hierarchical clustering, and confusion matrix between mental health categories identified by the four clinical scales' cutoffs and mental health categories identified by k-means clustering on the four clinical scales.
- **Figure S1**. Silhouette analysis displaying the optimal number of clusters.
- **Figure S2**. Output of random forest model: two grills displaying all variables tested for association with mental health status.
- **Table S4**. Results of univariate and multivariable regression models. The variables displaying the lowest AIC in the univariate logistic regressions are the seven most discriminating variables identified previously by random forest.
- **Figure S3A and Figure S3B.** Output of random forest model and Classification tree obtained using the polytomous version of the “risky behavior” variable.

**List S1.** List of standardized scales

The following are the standardized scales included in the multidimensional assessment.

- Generalized Anxiety Disorder Questionnaire (GAD-7, Spitzer et al., 2006), a validated instrument which measures anxiety symptoms with 7 self-rating items on a four-point scale, from 0 to 3. Cutoff points are 5 for mild, 10 for moderate and 15 for severe anxiety symptom levels. In this study, we adopted a cutoff score of 10, which was considered optimal in the original validation and subsequently used in several studies (Christensen et al., 2011; Vinogradova et al., 2022).
- Severity Measure for Depression – Adult (adapted from Patient Health Questionnaire – 9 [PHQ-9]), and Severity Measure for Depression – Child Age 11–17 (APA, 2015a; 2015b) adapted from the modified version for adolescents of PHQ-9 (Johnson et al., 2002). These two versions of the PHQ-9 were used to measure depressive symptoms, with 9 self-rating items on a four-point scale, from 0 to 3. Cutoff points are 5 for mild depression, 10 for moderate depression and at least 15 for moderate/severe and severe levels of depression. In this study, we adopted a cutoff score of 10, as suggested by a recent meta-analysis aimed at determining the accuracy of the PHQ-9 for screening to detect major depression (Levis et al., 2019).
- Somatic Symptom – Adult, and Somatic Symptom – Child Age 11-17 (APA, 2015a; 2015b), both adapted from the Patient Health Questionnaire Physical Symptoms (PHQ-15, Kroenke et al., 2002). These two self-administered versions of the PHQ-15 were used as a measurement tool of somatic symptom severity. The adult version consists of a list of 15 somatic symptoms, while in the Child Age version two items have been discarded. Each item is scored on a three-point scale ranging from 0 to 2. Cutoff points are 5 for mild somatic symptoms, 10 for moderate somatic symptoms and at least 15 for severe somatic symptoms. We used a cutoff score of 10, in line with previous studies (Kocalevent et al., 2013; Körber et al., 2011).
- PROMIS Emotional Distress – Anger – Short Form, and PROMIS Emotional Distress – Calibrated Anger Measure – Paediatric (APA, 2015a; 2015b). These two self-administered versions of the PROMIS were used as a measurement tool of the severity of anger. They include five questions on a five-point Likert scale, with a total score ranging between 5 and 25. Higher scores indicate higher severity of anger. The raw scores of the five questions are added to produce the total raw score, and the respective T score is checked. A T score <55 is considered normal; T score ≥55 and <60 indicates a mild level of anger; T score ≥60 and <70 indicates a moderate level of anger; and a T score ≥70 indicates a severe level of anger. In this study, we adopted the recommended mild cutoff T score of 55.
- Post Traumatic Growth Inventory - Short Form (PTGI-SF, Cann et al., 2010; Prati & Pietrantoni, 2014), a 10-item inventory with score range 0-50 that measures the extent to which individuals report positive life changes in the aftermath of a major life crisis. Five dimensions of PTGI-SF (new possibilities, relating to others, personal strength, appreciation of life, and spiritual change) are assessed with two items each. Items are rated on a 6-point Likert scale, from 0 (I did not experience this change as a result of my crisis) to 5 (I experienced this change to a very great degree as a result of my crisis). Higher scores indicate higher levels of post-traumatic growth.
- UCLA Loneliness Scale (UCLA, Russell, 1996), a 20-item scale designed to measure one’s subjective feelings of loneliness as well as feelings of social isolation. Participants rate each item as either O (“I often feel this way”, corresponding to a score of 3), S (“I sometimes feel this way”, corresponding to a score of 2), R (“I rarely feel this way”, corresponding to a score of 1), and N (“I never feel this way”, corresponding to a score of 0). The total score is the sum of all the items, with higher scores indicating greater feelings of loneliness.
- Connor-Davidson Resilience Scale 10 items (CD-RISC-10, Campbell-Sills & Stein, 2007). It consists of 10 statements describing different aspects of resilience. Each item is scored on a five-point scale ranging from 0 to 4, with 0 representing that the resilience statement is not at all true and a score of 4 indicating that the statement is true nearly all the time. The total score ranges from 0 to 40, with higher scores suggesting greater resilience.
- Fear of COVID-19 Scale, Italian version (Soraci et al., 2020), a seven-item scale that assesses the fear of COVID-19. The seven items are rated on a 5-point scale from 1 (strongly disagree) to 5 (strongly agree) with scores ranging from 7 to 35. The higher the score, the greater the fear of COVID-19.
- Sleep Problems Domain of the DSM-5 Self-Rated Level 1 Cross-Cutting Symptom Measure – Adult, and DSM-5 Self-Rated Level 1 Cross-Cutting Symptom Measure – Child Age 11-17 (APA, 2015a; 2015b). In these scales each item is rated on a five-point scale ranging from 0 to 4 (0 = None; 1 = Slight; 2 = Mild; 3 = Moderate; 4 = Severe) that measure the severity of the symptom. A score equal or above 2 in at least one of the items of the domain indicates the need of a more extensive evaluation.
- An adapted version of a selection of items included in the Risky Behavior Questionnaire for Adolescents (RBQ-A, Auerbach et al., 2018), a 20-item self-report instrument, which assesses broad-based engagement in risky behaviors in the past month. Each item is scored on a five-point scale ranging from 0 to 4 (0 = Never; 1 = Almost never, 1 time per month; 2 = Sometimes, 2/4 times per month; 3 = Quite often, 2/3 times per week; 4 = Often, 4 or more times per week).

**References:**

APA – American Psychiatric Association. Scale di valutazione adulti, 2015a, Raffaello Cortina Editore.

APA – American Psychiatric Association. Scale di valutazione soggetti 6-17 anni, 2015b, Raffaello Cortina Editore.

Auerbach RP, Gardiner CK. Behav Res Ther. 2012;50(10):596-603.

Campbell-Sills L, Stein MB. J Trauma Stress. 2007 Dec;20(6):1019-28.

Cann A, Calhoun LG, Tedeschi RG, Taku K, Vishnevsky T, Triplett KN, Danhauer SC. Anxiety Stress Coping. 2010;23(2):127-37.

Christensen H, Batterham PJ, Grant JB, Griffiths KM, Mackinnon AJ. BMC Med Res Methodol. 2011;11:154.

Johnson JG, Harris ES, Spitzer RL, Williams JB. J Adolesc Health. 2002;30(3):196-204.

Kocalevent RD, Hinz A, Brähler E. BMC Psychiatry. 2013;13:91.

Körber S, Frieser D, Steinbrecher N, Hiller W. J Psychosom Res. 2011;71(3):142-147.

Kroenke K, Spitzer RL, Williams JB. Psychosom Med. 2002;64(2):258-266.

Levis B, Benedetti A, Thombs BD; DEPRESsion Screening Data (DEPRESSD) Collaboration. 2019;365:l1781]. BMJ. 2019;365:l1476.

Prati G, Pietrantoni L. J Loss Trauma. 2014;19:1, 12-22,

Russell DW. J Pers Assess. 1996;66(1):20-40.

Soraci P, Ferrari A, Abbiati FA, Del Fante E, De Pace R, Urso A, Griffiths MD. Int J Ment Health Addict. 2020 May 4:1-10.

Spitzer RL, Kroenke K, Williams JB, Löwe B. Arch Intern Med. 2006;166(10):1092-1097

Vinogradova VV, Kivite-Urtane A, Vrublevska J, Rancans E. Medicina (Kaunas). 2022;58(9):1163.

**Table S1.** Frequencies of risky behaviors in the last month

|  | Never | Almost never | Sometimes | Quite often | Often |
| --- | --- | --- | --- | --- | --- |
|  | *n (%)* | *n (%)* | *n (%)* | *n (%)* | *n (%)* |
| Have you made attempts hurt yourself deliberately (e.g. cuts, burns) without intention to kill yourself? | 5070 (70.9) | 1019  (14.3) | 577  (8.1) | 279  (3.9) | 201  (2.8) |
| Have you purged or binged? | 4461  (62.4) | 1361  (19.0) | 666  (9.3) | 405  (5.7) | 253  (3.5) |
| Have you driven (a bicycle, a moped, and/or a car)  recklessly (e.g., at fast speeds, under the influence of a substance)? | 5652  (79.1) | 768  (10.7) | 345  (4.8) | 209  (2.9) | 172  (2.4) |
| Have you been binge drinking and/or drinking to get drunk? | 4131 (57.8) | 1317  (18.4) | 1133  (15.9) | 413  (5.8) | 152  (2.1) |
| Have you used cannabis? | 5955  (83.3) | 645  (9.0) | 277  (3.9) | 130  (1.8) | 139  (1.9) |

**Table S2.** Standardized tools and risky behaviors for gender variable, ‘Other’ category (n = 106)

| *Standardized tools* | Mean (SD) | Above cutoff |
| --- | --- | --- |
| PHQ-9 | 14.35 (8.45) | 67.0% |
| PHQ-15 | 11.28 (7.14) | 61.3% |
| PROMIS (T-score) | 56.34 (14.88) | 51.9% |
| GAD-7 | 11.63 (6.69) | 61.3% |
| Fear of COVID-19 | 14.27 (5.87) |  |
| PTGI-SF | 14.16 (12.07) |  |
| CD-RISC-10 | 13.99 (10.13) |  |
| UCLA | 29.36 (15.46) |  |
|  | Yes(%) | No(%) |
| *DSM5 Cross-Cutting Symptoms*-  Sleep Problems | 49.1 | 50.9 |
| *Risky behaviors^d^* | Yes(%) | No(%) |
| Self-harm | 58.5 | 41.5 |
| Binge eating | 52.8 | 47.2 |
| Reckless driving | 20.8 | 79.2 |
| Binge drinking | 41.5 | 58.5 |
| Cannabis use | 15.1 | 84.9 |

*Note:* PHQ-9: Severity Measure for Depression adapted from Patient Health Questionnaire- 9, PHQ-15: Somatic Symptom adapted from the Patient Health Questionnaire Physical Symptoms, PROMIS: PROMIS Emotional Distress – Anger, GAD-7: Generalized Anxiety Disorder Questionnaire, PTGI-SF: Post Traumatic Growth Inventory -short form, CD-RISC-10: Connor-Davidson Resilience Scale, UCLA: UCLA Loneliness Scale.

**Table S3A.** Mean scores of the four clinical scales by mental health group, identified by hierarchical clustering

|  | **PHQ-9**  Mean (SD) | **GAD-7**  Mean (SD) | **PROMIS**  Mean (SD) | **PHQ-15**  Mean (SD) |
| --- | --- | --- | --- | --- |
| **Good mental health** | 4.92 (3.76) | 5.55 (4.17) | 40.96 (6.26) | 4.71 (3.87) |
| **Poor mental health** | 12.83 (5.98) | 12.63 (5.01) | 61.46 (8.04) | 10.77 (5.70) |

**Table S3B.** Confusion matrix between mental health categories identified by the four clinical scales' cutoffs and mental health categories identified by k-means clustering on the four clinical scales

| **CONFUSION MATRIX** |  | |
| --- | --- | --- |
|  | **K-means clustering classification** | |
| **Cutoff classification** | *Good mental health (n)* | *Poor mental health (n)* |
| **PHQ-9** |  |  |
| *Good mental health (n)*  *Poor mental health (n)* | 3564 | 485 |
|  | 476 | 2514 |
| **GAD-7** |  |  |
| *Good mental health (n)* | 3384 | 373 |
| *Poor mental health (n)* | 656 | 2626 |
| **PROMIS** |  |  |
| *Good mental health (n)* | 3504 | 604 |
| *Poor mental health (n)* | 536 | 2395 |
| **PHQ-15** |  |  |
| *Good mental health (n)* | 3701 | 929 |
| *Poor mental health (n)* | 339 | 2070 |

Hierarchical clustering shows that 2 groups are clearly identifiable: A “good mental health” group displaying lower scores on all four scales and a “poor mental health” group displaying higher scores on all four scales. This justifies the creation of a categorical variable coded 0 for good mental health and 1 for poor mental health, derived on the 2 clusters imposed with k-means clustering and based, in turn, on the hierarchical clustering results. A confusion matrix was used to evaluate the accuracy between the classification based on k-means clustering and the classification based on the cutoffs of standardized scales.

**Figure S1.** Silhouette analysis


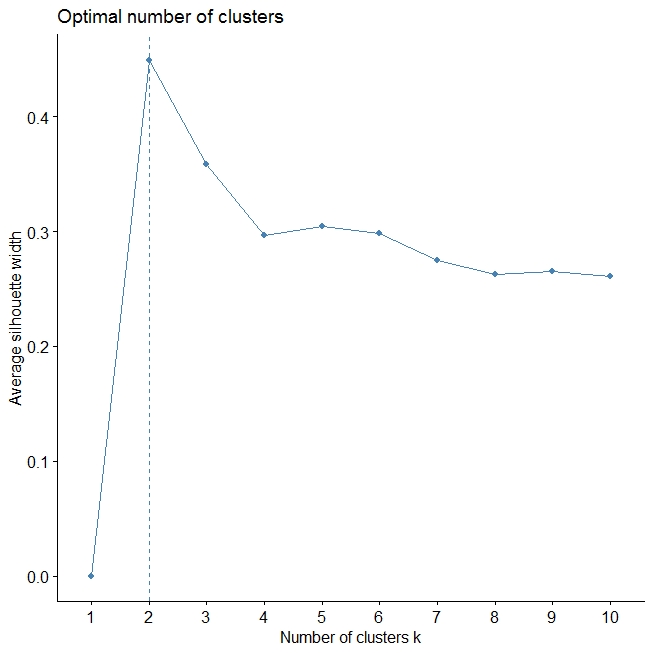


The silhouette analysis identifies the optimal number of clusters to separate the data into, based on the clinical scales scores, and further confirms that we can identify two well separated clusters based on mental health status.

**Figure S2.** Random forest

**
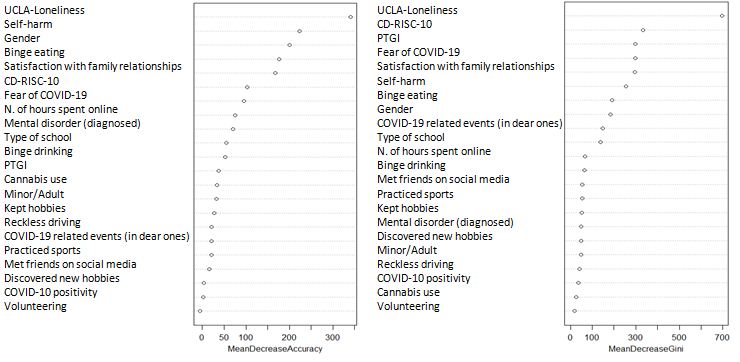
**

*Note*: accuracy of Random forest model: 80.6%

The ranking is based on two indexes measuring variable importance: Mean Decrease Accuracy and Mean Decrease Gini. The variables positioned at the top of the grills are the most discriminating between the good and poor mental health. The selection of the seven best discriminating variables was made according to both indexes.

**Table S4.** Logistic regressions. Univariate logistic models for each variable and multiple logistic models including the most significant variables

| **UNIVARIATE LOGISTIC REGRESSIONS** | Odds ratio­­­^e^ | Confidence Interval^e^ | *p*-value | AIC |
| --- | --- | --- | --- | --- |
| Gender | 0.25 | 0.23 – 0.28 | **<2E-16** | 8940.9 |
| Minor/Adult^a^ | 0.72 | 0.65 – 0.79 | **1.35E-11** | 9561.6 |
| Education^b^ |  |  | **<2E-16** | 9487.2 |
| *University* *vs* *Scientific/Grammar h.s.* | 0.51 | 0.43 – 0.60 | **<1.20E-15** |  |
| *University* *vs Technical inst.* | 0.68 | 0.57 – 0.81 | **2.83E-08** |  |
| *University* *vs Professional inst.* | 0.85 | 0.67 – 1.08 | .462 |  |
| *Technical inst. vs* *Scientific/Grammar h.s.* | 0.75 | 0.64 – 0.87 | **1.21E-05** |  |
| *Professional inst. vs* *Scientific/Grammar h.s.* | 0.60 | 0.47 – 0.75 | **2.64E-08** |  |
| *Professional inst. vs Technical inst.* | 0.80 | 0.63 – 1.01 | .073 |  |
| UCLA | 1.09 (${OR}_{std}=$ 3.14) | 1.08 – 1.09 (${CI}_{std}=$ 2.95 – 3.34) | **<2E-16** | 7913.7 |
| Fear of COVID-19 Scale | 1.09 (${OR}_{std}=$ 1.53) | 1.08 – 1.10 (${CI}_{std}=$ 1.45 – 1.61) | **<2E-16** | 9312 |
| CD-RISC-10 | 0.93 (${OR}_{std}=$ 0.57) | 0.93 – 0.94 (${CI}_{std}=$ 0.544 – 0.60) | **<2E-16** | 9125.3 |
| PTGI-SF | 1.01 (${OR}_{std}=$ 1.05) | 1.0003 – 1.01 (${CI}_{std}=$ 1.004 – 1.10) | **.035** | 9603.2 |
| Mental disorder (lifetime diagnosis) | 3.69 | 3.12 – 4.38 | **<2E-16** | 9351.4 |
| COVID-19 positivity | 1.07 | 0.93 – 1.22 | .361 | 9606.8 |
| COVID-19 related events  (in dear ones) ^b,c^ |  |  | **2.31E-16** | 9535.7 |
| *4 vs 1* | 1.75 | 1.44 – 2.13 | **1.67E-13** |  |
| *4 vs 2* | 1.43 | 1.20 – 1.70 | **4.12E-07** |  |
| *4 vs 3* | 1.07 | 0.87 – 1.32 | 1 |  |
| *2 vs 1* | 1.23 | 1.04 – 1.45 | **.008** |  |
| *3 vs 1* | 0.64 | 1.34 – 2.01 | **9.60E-10** |  |
| *3 vs 2* | 1.34 | 1.11 – 1.61 | **2.11E-04** |  |
| Satisfaction with family relationships | 0.68 (${OR}_{std}=$ 0.45) | 0.66 – 0.70  (${CI}_{std}=$ 0.43 – 0.48) | **<2E-16** | 8721.9 |
| *In the last month* |  |  |  |  |
| Binge drinking | 1.68 | 1.53 – 1.85 | **<2E-16** | 9494.9 |
| Self-harm | 5.65 | 5.05 – 6.34 | **<2E-16** | 8621.4 |
| Binge eating | 4.15 | 3.75 – 4.60 | **<2E-16** | 8822.3 |
| Cannabis use | 1.75 | 1.47 – 2.09 | **7.04E-10** | 9569.3 |
| Reckless driving | 1.22 | 1.09 – 1.37 | **6.14E-04** | 9595.9 |
| N of hours spent online ^d^ | 2.37 | 2.12 – 2.65 | **<2E-16** | 9371.5 |
| *During lockdown* |  |  |  |  |
| Practices sports | 0.72 | 0.65 – 0.79 | **1.92E-11** | 9562.2 |
| Met friends on social media | 0.91 | 0.83 – 0.998 | **.046** | 9603.6 |
| Kept hobbies | 0.53 | 0.48 – 0.59 | **<2E-16** | 9447.4 |
| Discovered new hobbies | 0.92 | 0.83 – 1.02 | .131 | 9605.3 |
| Volunteering | 0.86 | 0.70 – 1.04 | .125 | 9605.2 |
| **MULTIPLE LOGISTIC REGRESSION** |  |  |  |  |
| UCLA | 1.06 (${OR}_{std}=$ 2.24) | 1.05 – 1.07 (${CI}_{std}=$ 2.09 – 2.42) | **<2E-16** |  |
| Self-harm | 3.03 | 2.63 – 3.48 | **<2E-16** |  |
| Gender | 0.32 | 0.28 – 0.36 | **<2E-16** |  |
| Binge eating | 2.52 | 2.22 – 2.85 | **<2E-16** | 6424.6 |
| Satisfaction with family relationships | 0.78 (${OR}_{std}=$ 0.61) | 0.76 – 0.81 (${CI}_{std}=$ 0.57 – 0.65) | **<2E-16** |  |
| CD-RISC- 10 | 0.98 (${OR}_{std}=$ 0.84) | 0.97 – 0.99 (${CI}_{std}=$ 0.78 – 0.89) | **9.96E-08** |  |
| Fear of COVID-19 Scale | 1.06 (${OR}_{std}=$ 1.36) | 1.04 – 1.08 (${CI}_{std}=$ 1.28 – 1.46) | **<2E-16** |  |

*Note*: Dependent variable "mental health": 0 = good mental health, 1 = poor mental health. ^a^ 0 = Minor, 1 = Adult;

^b^ Bonferroni-correction for multiple comparisons was carried out for this regressor; ^c^ 1 = none, 2 = dear one(s) with COVID-19 positivity, 3 = dear one(s) being hospitalized (survived), 4 = death of dear one(s); ^d^ 0 = less than 5, 1 = 5 or more; ^e^ ${OR}_{std}, {CI}_{std}$: standardized odds ratio and confidence intervals for continuous variables.

For each of the seven variables identified, we see that: i) students with self-harm behaviors had a 5.65 (95% CI: 5.05 – 6.34) times higher probability to fall into the “poor mental health” category, compared to those not presenting such risky behaviors (p < 2e-16); ii) students with binge eating behaviors had a 4.15 (95% CI: 3.75 – 4.60) times higher probability to fall into the “poor mental health” category, compared to those without such behaviors (p < 2e-16); iii) males had a 75% (95% CI: 72% - 77%) lower probability to fall into the “poor mental health” category, compared to females (p < 2e-16); iv) each extra standard deviation in the scale measuring loneliness determined a 3.14 (95% CI: 2.95– 3.34) times higher probability to fall into the “poor mental health” category (p < 2e-16); v) each extra standard deviation in the scale measuring the fear of COVID-19 suggested a 53% (95% CI: 45% - 61%) increase in the probability to fall into the “poor mental health” category (p < 2e-16); vi) each extra standard deviation in the scale measuring satisfaction with family relationships suggested a 55% (95% CI: 52% - 57%) decrease in the probability to fall into the “poor mental health” category (p < 2e-16); vii) each extra standard deviation in the scale measuring resilience suggested a 43% (95% CI: 40% - 46%) decrease in the probability to fall into the “poor mental health” category (p < 2e-16).

Even though not among the seven most significant variables, we noted that the lifetime diagnosis of any mental health disorder was associated to a 3.69 (95% CI: 3.12 – 4.38) times higher probability to fall into the “poor mental health” category; moreover, students who spent five or more hours online daily had a 2.37 (95% CI: 2.12 – 2.65) times higher probability to fall into the “poor mental health” category.

Finally, the multivariable model confirmed the results of the random forest. The AIC was considerably lower than any of the univariate models and all seven variables were highly significant.

**Figure S3A**. Random forest obtained using the polytomous version of the “risky behavior” variable


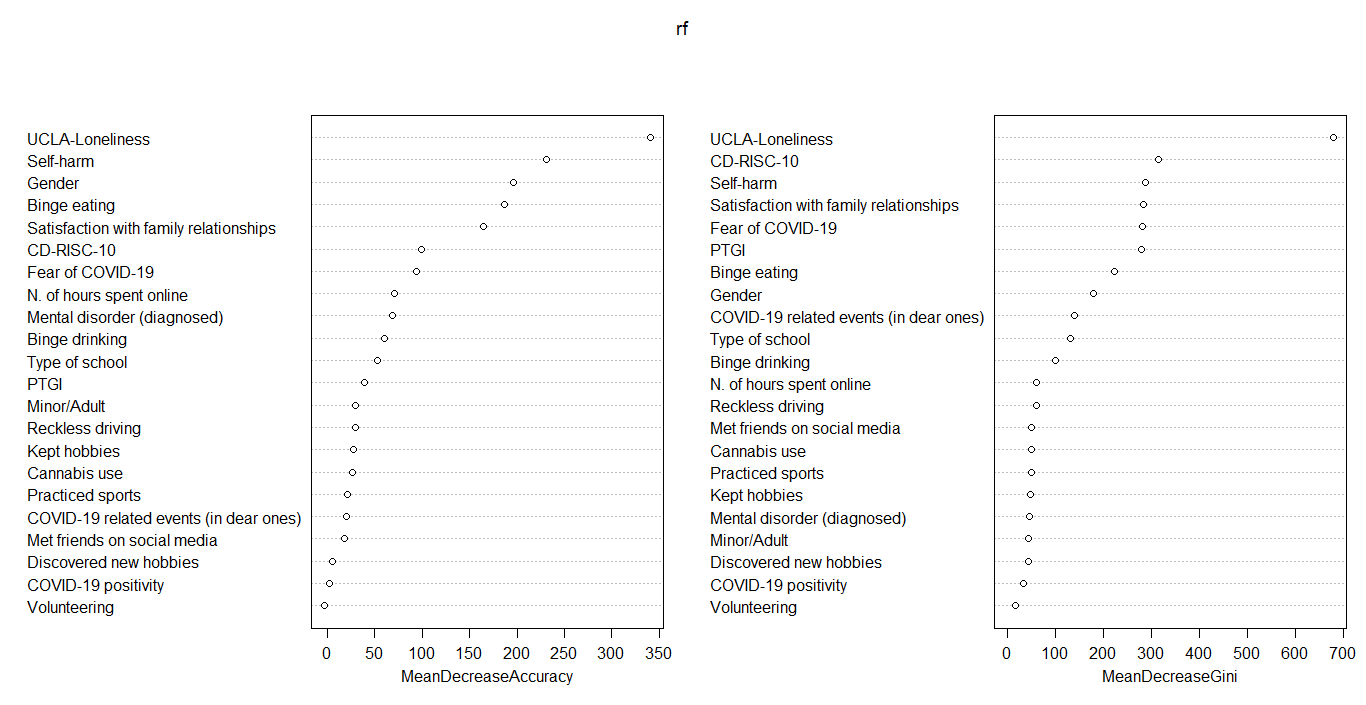


**Figure S3B.** Classification tree obtained using the polytomous version of the “risky behavior” variable


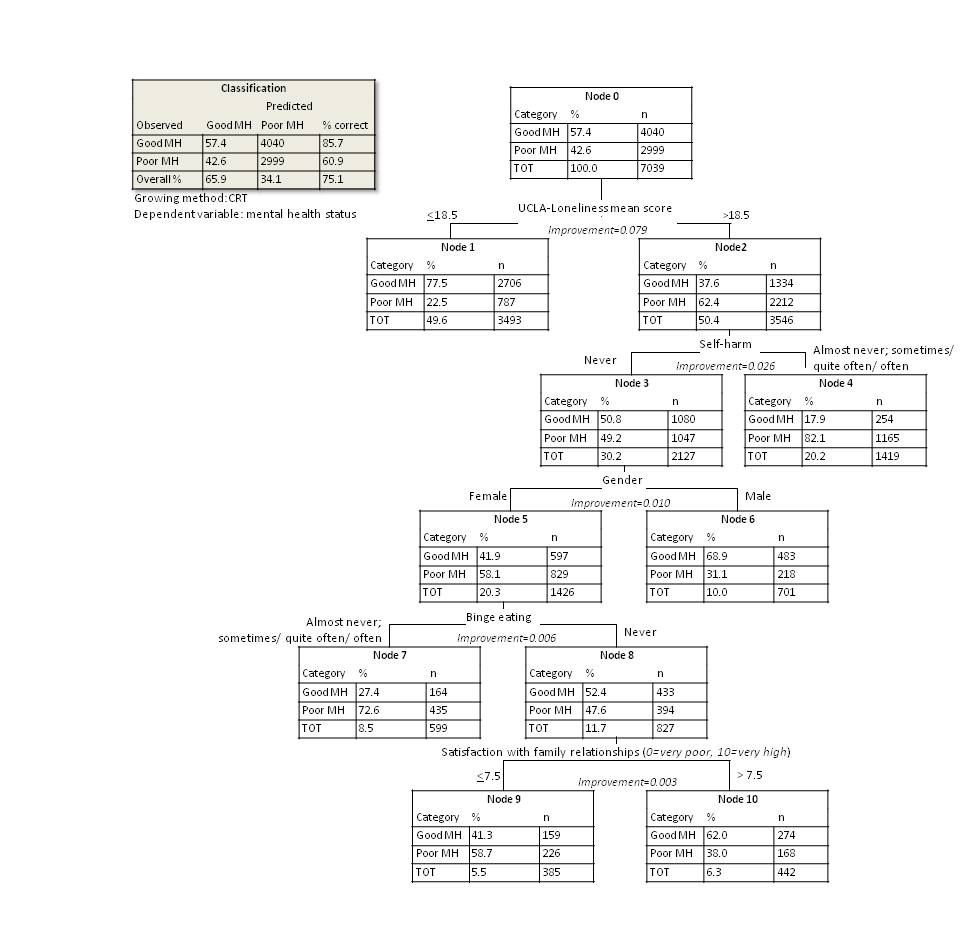

Supplement: Supplementary file 1 — Additional file 1: List S1. List of standardized scales. A list of the standardized scales included in the assessment. Table S1. Frequencies of risky behaviors, in the last month. Table S2. Standardized tools and risky behaviors for gender variable, ‘Other’ category. Table S3. A and B Mean scores of the four clinical scalesby mental health group, where groups are identified by hierarchical clustering, and confusion matrix between mental health categories identified by the four clinical scales' cutoffs and mental health categories identified by k-means clustering on the four clinical scales. Figure S1. Silhouette analysis displaying the optimal number of clusters. Figure S2. Output of random forest model: two grills displaying all variables tested for association with mental health status. Table S4. Results of univariate and multivariable regression models. The variables displaying the lowest AIC in the univariate logistic regressions are the seven most discriminating variables identified previously by random forest. Figure S3. A and B. Output of random forest model and Classification tree obtained using the polytomous version of the “risky behavior” variable. [file 13034_2023_615_MOESM1_ESM.docx]
